# Remotely Exploring Deeper-Into-Matter by Non-Contact Detection of Audible Transients Excited by Laser Radiation

**DOI:** 10.3390/s17122960

**Published:** 2017-12-20

**Authors:** Javier Moros, Inmaculada Gaona, J. Javier Laserna

**Affiliations:** UMALaserlab, Departamento de Química Analítica, Facultad de Ciencias, Universidad de Málaga, Jiménez Fraud 4th, 29010 Málaga, Spain; inmagfdz@uma.es (I.G.); laserna@uma.es (J.J.L.)

**Keywords:** laser-based sensor, acoustic emission, subsurface spectroscopy, concealed materials

## Abstract

An acoustic spectroscopic approach to detect contents within different packaging, with substantially wider applicability than other currently available subsurface spectroscopies, is presented. A frequency-doubled Nd:YAG (neodymium-doped yttrium aluminum garnet) pulsed laser (13 ns pulse length) operated at 1 Hz was used to generate the sound field of a two-component system at a distance of 50 cm. The acoustic emission was captured using a unidirectional microphone and analyzed in the frequency domain. The focused laser pulse hitting the system, with intensity above that necessary to ablate the irradiated surface, transferred an impulsive force which led the structure to vibrate. Acoustic airborne transients were directly radiated by the vibrating elastic structure of the outer component that excited the surrounding air in contact with. However, under boundary conditions, sound field is modulated by the inner component that modified the dynamical integrity of the system. Thus, the resulting frequency spectra are useful indicators of the concealed content that influences the contributions originating from the wall of the container. High-quality acoustic spectra could be recorded from a gas (air), liquid (water), and solid (sand) placed inside opaque chemical-resistant polypropylene and stainless steel sample containers. Discussion about effects of laser excitation energy and sampling position on the acoustic emission events is reported. Acoustic spectroscopy may complement the other subsurface alternative spectroscopies, severely limited by their inherent optical requirements for numerous detection scenarios.

## 1. Introduction

The inspection of suspicious objects poses specific, and often, difficult analytical challenges. The use of sensors to operate in real time, in situ, and contactless, but rapidly and with appropriate sensitivity is often necessary in an *IED* (improvised explosive device) scenario due to the boundary conditions that characterize it. Laser-based sensors with spectroscopic detection methods, including laser photolysis/laser-induced fluorescence, laser-induced breakdown spectroscopy, as well as vibrational spectroscopies, like Raman and coherent anti-Stokes Raman scattering, pool those requirements and have been tested over recent years for explosives detection [1,2]. However, most of these laser-spectroscopy combinations unfold their full potential for the remote, but surface, detection of contaminations of explosives [3], since acquiring information of the inner side implies the prior invasion of the outer layer.

Although there are spectroscopic techniques that are able to “see inside through barriers concealing” by exploiting changes in light properties such as optical absorption [4,5], THz imaging at the transmission and reflectivity modes [6,7], fluorescence [8], and elastic scattering based on the spatial offset scheme [9,10,11], they have several inherent optical requirements that limit the accessible depths and consequently the obtainable signal. Deeper probing in some matrices is therefore not addressable by these conventional approaches.

A complementary strategy to address these constraints lies on the use of laser generated surface acoustic waves (*SAW*s) [12,13,14,15,16]. Laser light at a surface, may generate thermally-induced transient stresses that cause the strengthening of its vibration. During the laser-matter interaction processes the surface usually gains momentum and energy, and hence, various types of thermo-elastic confined waves, bulk and acoustic, are launched from the illuminated area [17,18,19,20,21,22]. The mechanisms behind the generation of *SAW*s depend on the parameters (wavelength (*λ*), pulse duration (*τ*), energy (*E*), and focusing geometry) of laser light deposited on and absorbed in the surface layer of the solid, as well as on the properties of the latter (especially on its absorption coefficient α at λ radiation wavelength and the ratio of the thermal expansion coefficient, *β*, to the specific heat of the solid specimen, *c_p_*) [23]. All these variables together characterize the physical phenomena leading to the conversion of radiation to acoustical energy [24].

The excitation of acoustic waves by the interaction of laser radiation with matter has been experimentally and theoretically studied in detail for gases [25,26], liquids [26,27], and solids [28,29]. Particularly, the acoustic waves generated by laser pulses directed onto solid surfaces have become invaluable tools for non-contact monitoring of laser-based processes [30] as welding [31,32], cladding [33], cutting [34], and cleaning [35], as well as to anticipate any malfunction of a material during its manufacturing [36], to cite some examples. The generation of acoustic waves has been also extensively investigated in parallel with outcropping of other optical phenomena like laser-induced plasmas of solid surfaces. In this ablative domain, easily obtained from a pulsed laser in the focus of a lens, when the solid is subjected to thermal and/or mechanical changes, the pattern of sound generation may become complex since nonlinear acoustic emissions arise due to the sudden release of thermal energy [37,38,39,40]. In any case, these laser-generated acoustic transients have become a promising tool for monitoring and control the ablation process from fundamental properties like the stress power (laser energy coupling to the solid target) [41], the plasma formation mechanism and its evolution dynamics [42,43] and the ablation rate [44,45,46], as well as for standardizing the retrieved analytical signal via normalization of optical emissions [47,48].

Be that as it may, in the case of solid systems, up until now most of the theoretical and experimental research has been mainly focused on the acoustic response of homogeneous single-layer materials [44,49,50]. In contrast, the examination and characterization of bi- and multi-layered materials through their transient acoustic waves have been scarcely tackled [51,52,53,54]. Furthermore, mostly acoustic methods are based on structure borne emission monitoring rather than airborne acoustic emissions, which can be subjected to more disturbances and thus are considered less reliable. In short, a critical juncture that opens the door to investigate the possibility of using laser-generated acoustic transients for a distant screening of multi-component systems; the boundary conditions entailing an *IED* scenario; remote-for-safety inspection.

Within this context, a simple approach for retrieving analytical information from contents in non-transparent containers through the acoustic field in the far zone of the antenna originated at the laser ablated area has been proposed. The current work describes the contribution of the audible responses retrieved from remotely laser-excited bi-layer systems with a solid on one hand and, a gas, a liquid, or another solid on the other. The characteristics of acoustic spectroscopy make the proposed technology a competitive method for inspecting a wide variety of packaging and samples. Thus, from an application perspective, remote audible responses may be ideally suited to defense and security applications, mainly for countering *IED*s. The acoustic measurements of target content can aid in the detection, diagnosis, and positive characterization of a threat towards effective making decisions.

## 2. Materials and Methods

### 2.1. Laser-Based Acoustic Sensor

While the design of a field deployable sensor is pursued, at this preliminary phase of the research a laboratory scale prototype has been considered. The acoustic sensor consisted of a Q-switched Nd:YAG (neodymium-doped yttrium aluminum garnet) laser (532 nm, 50 Hz, 70 mJ·pulse^−1^, 13 ns pulse width, flat top hat distribution intensity) as excitation source. Laser pulses at low repetition rate (1 Hz) were directly focused on the target surface through a 75 mm focal length plane-convex quartz lens, thereby reaching a spot diameter of ca. 200 μm. Acoustic signals launched into ambient atmosphere were captured using an ultra-linear measurement condenser (20 Hz–20 kHz, ECM8000 model) microphone located coaxial to the source-receiver path at a fixed distance of 50 cm. Transduced sounds were digitized using a Roland UA-55 Quad-capture audio interface at the standard sampling rate of 96 kHz. For the standoff acoustic sensor, these components will be replaced by a telescope to focus the laser pulses at specific analysis distances, and a high performance unidirectional microphone perfectly suited to the requirements of security and surveillance applications and capable of picking up sounds from a distance of 100 m and beyond in noisy environments.

Targets were housed inside a purpose-built hemi-anechoic chamber (35 × 35 × 35 cm^3^, *L* × *W* × *H*) in a rotating platform on top of a *Z*-axis linear translation stage to refresh the sampling position into the target. Typical rectangular base cones were used to form the absorbing boundaries inside the chamber to reduce echoes as well as vibrations and noise contributions from outside. During operations, the temperature and relative humidity of the lab-room were real-time monitored (weather station PCE-FWS 20 model) at 25 ± 2 °C and 50 ± 3%, respectively. A total of sixty audible events (recording time was 60 s) were laser-stimulated in each of the acoustic trials. To diagnose the acoustic differences time-domain waveforms were resolved into frequency components spectra of data using fast Fourier transform (FFT) methods. It must be mentioned that, for signal processing, a frequency selective filter was designed; an amplitude high pass filter. This tool permitted frequency components with an amplitude value above a threshold (ten percent of amplitude of the dominant frequency) to pass unattenuated whereas scores of frequency components with amplitude below the threshold were smoothly brought down to zero.

The optical emission spectra of the plasma sources launched in parallel with the acoustic events were also gathered. The light emitted was collected orthogonally to the plasma expansion axis and collimated into a 1000 µm core fiber with a 8.7 mm confocal length lens. Collected light was then guided to the entrance (10 μm slit) of a *Czerny-Turner* spectrograph (303 mm focal length, *f*/4) fitted with a diffraction grating of 150 lines per mm and coupled to an intensified CCD detector (intensifier tube diameter of 25 mm). This configuration provided an effective spectral range covering from 185 nm to 780 nm with a spectral resolution of 0.88 nm. Plasma light was acquired using a 2 µs gate width at 1.7 µs delay from the external trigger input (zero-time position) supplied by the laser Q-switch output signal to the opening of the camera intensifier tube. Optical emission spectra were used in a preliminary step for labeling the target scenario into a particular class (stainless steel or polypropylene in the cases in question) so to collate the acoustic output from the unknown against a discrete set of acoustic spectra rather than with all the sound data stored in a spectral library.

### 2.2. Samples

A 118 mL chemical-resistant polypropylene (PP) bottle (1 mm wall thickness), and a pair of stainless steel (SS) (AISI 301 series grade) vessels (0.25 mm wall thickness), 350 mL and 250 mL, respectively, were decided as sample containers. The choice of these materials was motivated by the anatomy of an *IED*. The container is a basic part of an explosive device. It can be configured in countless designs ranging from the most simple to highly sophisticated in shape, size, and design. A lot of *IED*s are deployed concealed within innocent plastic-walled and steel-walled items, such as food containers for food or a soda can, for which the explosive deflagration creates sufficient pressure to rupture them. To emulate gas, liquid, and solid explosive, atmospheric air, tap water, and fine-grained sand (<4 mm) were considered as the concealed substances for acoustic tests. For all cases, containers were completely filled and tightly closed with their corresponding caps. No trials over different fill-levels in the containers were attempted.

Since experiments were conducted at laser-ablation and plasma-formation regimes, there was no doubt that radiation was absorbed at the containers surface. Thus, during the irradiation of a translucent semi-crystalline thermoplastic, like PP, and an opaque material, such as SS, optical penetration depth was expected a few tens of nanometers, significantly lesser than the containers walls thickness. The absorption of Nd:YAG-laser radiation (532 nm) for PP was about of 30% of the incident energy, whereas for SS was estimated at 50%. This absorptivity changes during the heating process. As energy is transferred to the materials, they heat up and as the temperature is elevated the amount of absorbed light may change. In our performance, since laser pulses impinged on the container surface during a short width of 13 ns that accurately repeated once per second, no pulse-to-pulse changes in the mechanical, thermal and optical conditions of the finite-dimensional impact area are expected.

## 3. Results and Discussion

### 3.1. Laser-Induced Acoustic Responses of Containers

**Figure 1:**
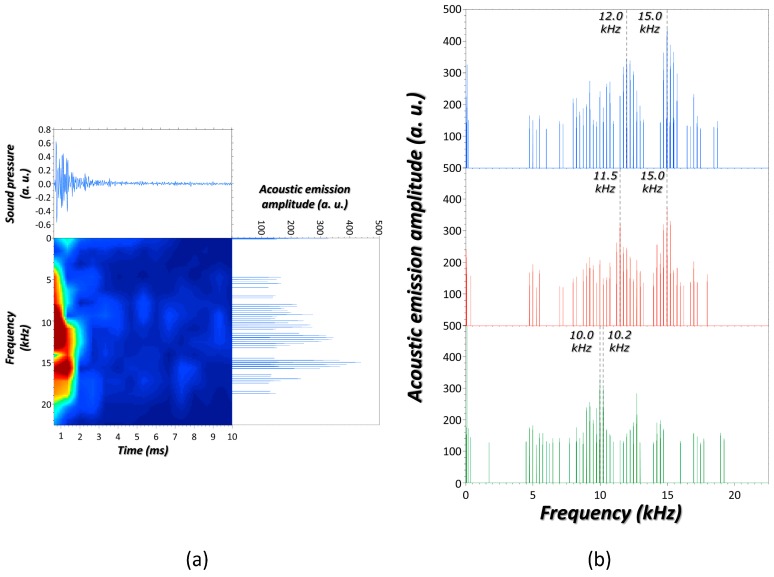
(**a**) Graphical representation of the laser-induced (40 mJ) acoustic response from the central position of the *generatrix* of an SS container (350 mL) filled with air; (**b**) Laser-induced acoustic spectra for the three containers considered containing air. From **top** to **bottom**: SS container (350 mL), SS container (250 mL), and polypropylene (PP) container. More details in the body of the text.

Figure 1a shows the acoustic signals recorded in the interaction of a laser pulse with an SS container filled with atmospheric air. The waveform discloses the variations in the magnitude of air pressure at discrete times. As seen, this is a short burst of sinusoidal components that drop exponentially to become depleted after about 3 ms. This series of non-periodic multiple pressure pulses represents the sequence of successive compressions and rarefactions of air molecules close to the laser-surface interaction area. In parallel, waveform is shown as a sound spectrum. As noticed, the Fourier transform of the waveform reveals the large number of distinct constituent frequencies of varying amplitudes. These frequencies define the particular envelope of modes with different hills and valleys for the interrogated target. The sound spectrum is disclosed synchronously through the acoustic spectrogram that visually displays the changes occurring over time in the energy of the frequency components. As observed, the more intense frequencies characterizing the interrogated system appear in the range between 4 kHz and 19 kHz, and captured below a time of 1.6 ms. Figure 1b compares the acoustic spectra in the frequency domain for the three evaluated containers filled of air. As seen, the crucial differences between acoustic spectra lie in a shift for the leading frequencies. SS containers reveal comparable prominent frequencies at roughly 12 kHz and 15 kHz, which significantly depart from the dominant frequencies featured at around 10 kHz in the spectrum of the PP bottle. These acoustic results are consistent with the elastic properties of materials. SS (elastic modulus ~180 GPa) is a rigid material that experiences a smaller deflection than PP (elastic modulus ~1.5–2 GPa) when the same uniform load (laser pulse) is applied to their surfaces. SS lattice is characterized by particles with strong forces of attraction for each other. These forces guide the rate with which particles return to their original position. Particles quickly regaining their resting position are ready to immediately vibrate. The more times the sound pressure varies from its equilibrium value, since numerous surface micrometric vibrations occur, the higher the frequencies [55].

A more detailed observation in Figure 1b draws out some differences to the frequency components within acoustic responses of the SS containers. Some dissipate whereas other new ones emerge. Besides, as seen, one of the dominant components within the acoustic signal of the small container (250 mL) shifts towards a lower frequency as compared to that in the spectrum of the larger one (350 mL). These events are argued on the basis of the influence of the geometry and boundary conditions of non-free surfaces on the launched elastic waves. Hence, despite containers being built from the same material, the mechanical behavior of these surfaces are subjected to distinct normal and tangent translational and rotational constraints [56,57].

Summarizing, laser-induced acoustic responses from rigid containers are able to materialize any difference associated to the microstructural vibration of their surfaces.

### 3.2. Laser-Induced Acoustic Responses of Concealed Materials

**Figure 2:**
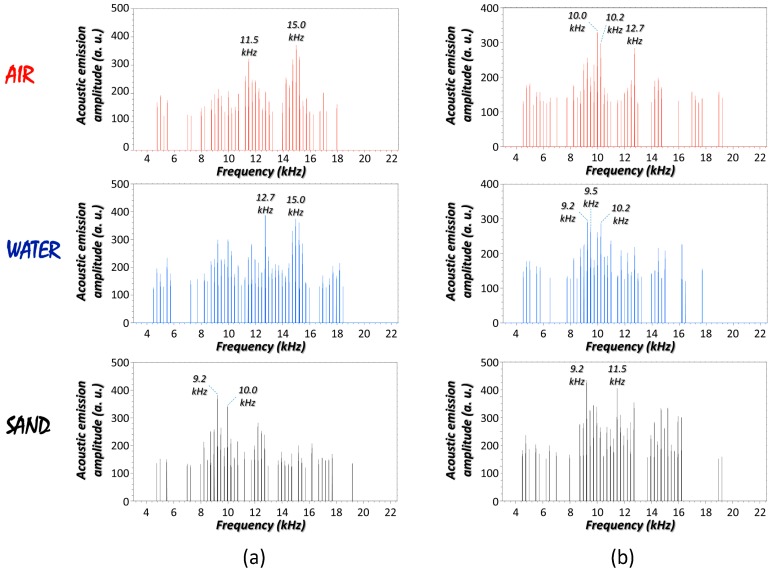
(**a**) Laser-induced (40 mJ) acoustic spectra for an SS container (250 mL) filled with different materials; (**b**) Laser-induced (40 mJ) acoustic spectra for a PP container filled with different materials. Sampling point was in both cases the central position of the container *generatrix*.

Acoustic responses retrieved from the laser-interrogation of the containers were scrutinized to determine the influence of materials filling with. To discuss about, Figure 2 shows the acoustic spectra gathered from the laser-excitation of different composite systems. As seen, for a given container, the frequency components and their amplitude change according the nature of its content. Specifically, the sound spectrum of the SS container (250 mL) filled with air (Figure 2a) shows strong peaks at roughly 11.5 kHz and 15.0 kHz. In contrast, the most intense frequency components for the container filled with water shifts towards 12.7 kHz, or at 9.2 kHz when filled with sand. Similarly, noticeable differences between the laser-induced acoustic emissions in the case of the PP container with different contents were also drawn (Figure 2b). Hence, the analysis revealed that the interior may play a dominant role in defining the external acoustic field. Results are justified on the basis of a defined boundary condition: the thickness of the outer layer is very thin compared to the inner content for the tested systems. Under this circumstance, the content influences the elastodynamic response emanating from the point source at the container surface. Acoustic signal comes from a unique vibrational pattern caused by the coexisting elastic forces of two neighboring layers laterally coupled. While it is expected that the thinner the container surface the more accentuated the influences of the content on the vibrational properties, further studies are needed in this direction.

With a view to quantify the similarity/difference between pairs of acoustic outcomes in Figure 2, the root mean square error (*RMSE*) score was calculated from Equation (1):

(1) $$RMSE=\sqrt{\frac{\sum_{i=1}^{N}{\left(\alpha_{i}-\beta_{i}\right)}^{2}}{N}}$$

where α and β correspond to the amplitude of each frequency for the whole bandwidth—*N*—that defines the acoustic spectra A and B, respectively. This common metric compares two acoustic signals by exerting a matching process dependent on the dominant features, both the frequency components and their amplitude. The interpretation of this quantitative score, which ranges from 0 to infinity, is as follows: the larger the *RMSE* value, the greater the differences between acoustic responses.

In the case of the SS container (Figure 2a), the *RMSE* values reported for the sand–water, sand–air, and water–air pairs were 9.08, 11.09, and 12.61, respectively. Similar scores were obtained in the case of the PP container (Figure 2b). These values, on average, were significantly higher than the statistic *RMSE* of 0.340 ± 0.016 from the comparison between ten independent repeated analyses of the same system—the PP container filled with sand. Hence, those results support the differences on the laser-induced acoustic response of a container depending on the content solid, liquid, or gas.

### 3.3. Effects of Operating Variables on the Acoustic Fingerprints

● *Laser Pulse Energy*

**Figure 3:**
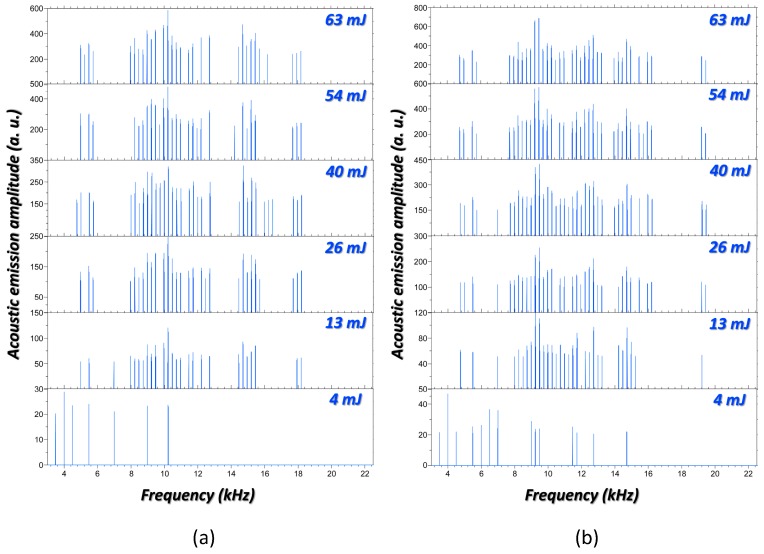
(**a**) Acoustic spectra induced at the bottom of the *generatrix* of an SS container (350 mL) filled with sand induced at varying laser pulse energy; (**b**) Acoustic spectra induced at the central position of the *generatrix* of an SS container (350 mL) filled with water induced at varying laser pulse energy.

As indicated in the introductory section, properties of laser radiation (pulse shape, wavelength, length, and energy) are key variables to characterize the launched acoustic waves [58,59]. Here, the effect of pulse energy on the acoustic frequency emissions during the laser-induced breakdown of systems was investigated. This was achieved by varying the input laser energy under the same focusing conditions. As case studies, the acoustic signals retrieved from the interrogation of two distinct scenarios at variable laser energy are shown in Figure 3.

Furthermore, to better evaluate the effects of the acting force on the acoustic signals, the *RMSE* values (*min-max* normalized) from the paired-comparison between data are pointed out in Table 1. From results it can be inferred that, for the interrogation of the same scenario, an increase in laser energy leads to different patterns on frequency components for the acoustic responses. The higher the energy gap the larger the differences. Numerous distinctive frequency components are easily detected between the acoustic signals generated at 4 mJ and 63 mJ, respectively. These results are consistent with the elastic properties of solids. As the magnitude of the force acting over the surface increases, it is deformed to a larger extent, thereby altering the rate to regaining of the equilibrium position. This surface displacement defines the vibrating pattern of the system that finally guides the sequences of compressions and rarefactions of air molecules circling the laser impact area.

Notwithstanding this, at high excitation regimes (54 mJ and 63 mJ) the variations experienced by surface vibrations resulting from the change of excitation energy seems to be less sensitive; a similar strain per unit area within the solid system arises. Maybe, this can be justified as a plateau for the maximum capacity of the material to elongate previous to the onset of permanent deformation. This circumstance leads to an increasing convergence of acoustic frequencies profiles as can be deduced from the decreasing of *RMSE* values in the last column of Table 1. Furthermore, the similar trends within *RMSE* values in both cases, A and B, reveal an analogy on the surface vibrational behavior, beyond that acoustic frequencies coming from each scenario are different because of their inherent characteristics. Notwithstanding this, laser energy is a crucial variable to consider since it influences the consistency of spectral information gathered during acoustic testing.

● *Sampling Point*

**Figure 4:**
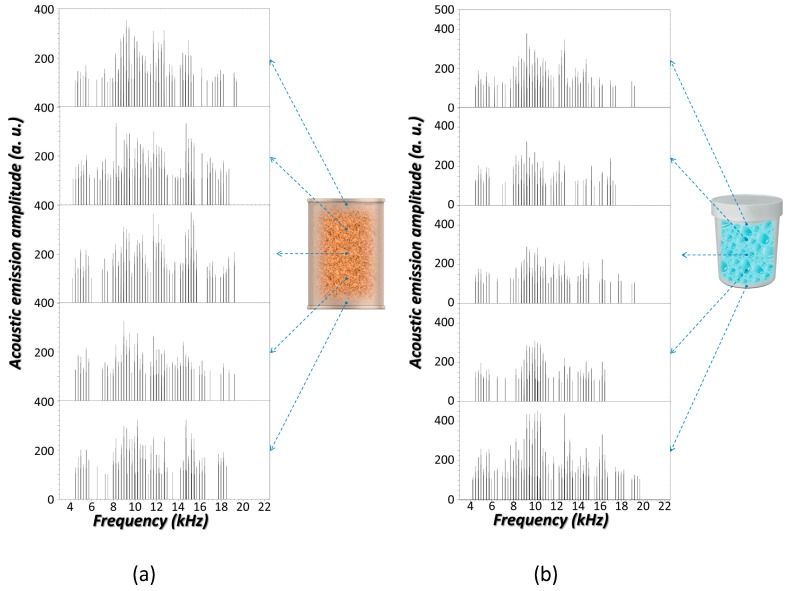
(**a**) Laser-induced (40 mJ) acoustic spectra for an SS container (350 mL) filled with sand at five different sampling positions along its *generatrix*; (**b**) Laser-induced (40 mJ) acoustic spectra for a PP container filled with water at five different sampling positions along its *generatrix*.

Acoustic emissions from the laser interrogation of systems were investigated to determine whether there would be any effect when the sampling position is varied. In Figure 4, the sequences of audible responses captured from spaced sampling points along the *generatrix* of two different containers filled with distinct materials are shown. As seen, for a particular scenario, acoustic spectra launched from different positions in the system are characterized by varied frequency components. Table 2 reports examples of the extent of discrepancies through *RMSE* scores from the paired-comparison between acoustic spectra from sampling points along the *generatrix* of a PP container when filled with different substances. As inferred from data, acoustic spectra significantly differ from one part of the container surface to another, no matter the underlying material. These results can be justified within the framework of the local elasticity for a non-free finite surface. Every point of the non-free surface is not equally supported by the same normal stiffness that represents their longitudinal (dilatational waves) and transversal (rotational waves) displacements and has different influences on the launched waves [60].

It is expected that the container wall satisfies a larger deformation at its central part while their displacements close to the container ends, principally at the base, are much lower. Thus, as can be extracted from data in Table 2, in general, the acoustic emissions from lowest positions at the container wall (*P1* and *P2*) significantly differ from that towards the rest of the positions (*P3*, *P4,* and *P5*). Furthermore, the inter-position acoustic differences seem to be more pronounced in the case of air as the content, as compared to water and sand. This circumstance can be justified by the density of the material. Highly-compacted sand particles confer similar stiffness at any site of the container as compared to air molecules. The greater the density of the content the lesser accentuated is the effect of the axial boundaries of the container wall on the vibrations and the ensuing acoustic emissions.

Despite this, differences between audible responses emerging from distinct points of the same system still amount significantly high to consider any acoustic signal as a unique “fingerprint” that unambiguously characterizes it. Data in Table 3 are ample proof that differences between acoustic responses for two different systems are comparable to those retrieved from varying positions of the same system. As an example, *RMSE* scores from the paired-comparison between acoustic spectra from a PP container with different contents are reported.

To deal with such uncertainties an acoustic emission spectroscopy longitudinal-profiling analysis of the system is suggested. An approach based on building an acoustic profile of five equidistant sampling points along the *generatrix* of each system was considered as a solution. Then, an “augmented fingerprint” shaped by concatenation of the acoustic emissions captured from the consecutive positions characterizes the system. By using this new tracking identifier, *RMSE* values of 8.96, 8.46, and 7.34 were calculated to the acoustic spectra comparison between air–water, air–sand, and water–sand contents in a PP container, respectively. While these scores are numerically similar to those attained for the comparison between acoustic emissions from single positions (Table 2) they should be differently interpreted. Indeed, they punctuate a larger divergence between systems of almost 20% since they are computed from a five times greater number of variables.

From the use of this new identifier based on a longitudinal-profiling approach for picking up sounds from a target, several important sensing parameters of the methodology can be defined. An accuracy of 100% on identification of content in PP and SS containers was validated by cross-checking acoustic outputs against a database consisted of the acoustic pattern of each container with their three variations associated to the contents, totaling nine different patterns. The precision of the sensing system to give the same acoustic pattern when repetitively measuring the same scenario under the same conditions, expressed as relative standard deviation (*RSD*) was 4%. In agreement with the approach proposed for identification purposes of non-seen contents, from results, a highly specific sensing has been attained. Sensor has derived an acoustic fingerprint completely specific to each a single scenario. Assuming that laser-generated acoustic patterns are influenced by the laser pulse properties (wavelength, duration, energy), the system characteristics (container, content, and their inherent qualities), and the conditions during the operation of the sensor (the target-to-sensor distance, environmental conditions of temperature and moisture...) it is difficult to stem the sensitivity spreading to the measurements. This is why, the sensitivity of the prototyped sensing system was defined as the sensitivity of the microphone =−60 dB; saying the microphone is producing a signal 60 dB below the one volt reference. Note that the higher the negative dB, the lower the sensitivity. The threshold for the minimum differentiable acoustic signal here could be established as the maximum thickness of the container wall that allows evidencing any content at a specified level of confidence. In this connection, it is expected that the thicker the container wall the higher the limit of detection (*LOD*). If the thickness of the container wall is quiet large itself this methodology would not be so successful. Despite this, results encourage to continue towards the design of a field-deployable laser-based device for in-situ and real-time acoustic inspection of containers and non-see contents discrimination.

## 4. Conclusions

The feasibility of using laser-induced acoustic signals to characterize the content of non-transparent containers has been demonstrated. The acoustic spectroscopy may yield analytical information which would be otherwise difficult or impossible to obtain with other subsurface spectroscopies, insofar as its propitious boundary conditions concur. It was shown that the microstructural vibrations forced on the surface of stainless steel and polypropylene containers filled with different materials (air, water, and sand) disturb the surrounding air, creating specific acoustic waves that move outward from the point of the laser impact. Notable differences have been detected in the acoustic measurements, namely due to the dependence of acoustic frequency components on laser energy dosage and on the longitudinal sampling point. Notwithstanding this, those differences can be usefully exploited towards the acoustic specificity of the systems. A longitudinal-profiling approach has been suggested for building an exclusive acoustic fingerprint of the system. Then, the identification capability would be based upon comparing the acoustic profile of the unknown system against an acoustic profiles database on board the device.

As it has been proven, the experimental evidences given can be considered as proof of concept on the performance of the sensor to identify three well-differentiated materials. However, much research still remains. Research to quantify the critical thickness for surfaces that allows collecting subsurface analytically useful acoustic information, as well as on robustness and ruggedness of acoustic signals from irregularly-shaped, cracked, and/or having-imperfections containers, should be conducted. Trials to assess sensing ability to discriminate between materials with similar but distinct acoustic permeability (different solids, liquids, and gases), and for categorizing complex systems (a container filled with 2–3 materials) should also be undertaken.

Furthermore, since this spectroscopy may provide orthogonal data to further increase accuracy and sensitivity, design of data fusion parametric approaches for simultaneous information from laser-induced optical emission spectroscopy should be welcome.

## Figures and Tables

**Table 1:** Root mean square error (*RMSE*) scores on the fidelity/distortion judgment between acoustic responses induced at variable laser pulse energy.

		***Normalized RMSE***					
	**Laser Energy (mJ)**	**4**	**13**	**26**	**40**	**54**	**63**
***Scenario A***	**4**	0.0000	0.1925	0.4369	0.6727	0.8856	1.0000
	**13**		0.0000	0.2807	0.5799	0.7785	0.8548
	**26**			0.0000	0.5444	0.7103	0.7290
	**40**				0.0000	0.4281	0.6214
	**54**					0.0000	0.5745
	**63**						0.0000
	**Laser Energy (mJ)**	**4**	**13**	**26**	**40**	**54**	**63**
***Scenario B***	**4**	0.0000	0.1524	0.3636	0.6512	0.8483	1.0000
	**13**		0.0000	0.3400	0.5668	0.7335	0.8824
	**26**			0.0000	0.4649	0.7983	0.8374
	**40**				0.0000	0.6415	0.7553
	**54**					0.0000	0.2666
	**63**						0.0000

**Table 2:** Root mean square error (*RMSE*) scores on the fidelity/distortion judgment between laser-induced acoustic responses from different positions of a PP container.

*RMSE*							
Content		*Sampling Point*	*P1*	*P2*	*P3*	*P4*	*P5*
***Air***	***(top)***	***P5***					0
		***P4***				0	6.10
	***(center)***	***P3***			0	6.12	6.18
		***P2***		0	9.83	10.12	8.48
	***(bottom)***	***P1***	0	9.14	11.97	12.66	10.77
***Water***	***(top)***	***P5***					0
		***P4***				0	5.99
	***(center)***	***P3***			0	6.20	6.45
		***P2***		0	5.75	6.47	6.84
	***(bottom)***	***P1***	0	9.05	8.84	10.52	9.09
***Sand***	***(top)***	***P5***					0
		***P4***				0	5.92
	***(center)***	***P3***			0	7.29	6.78
		***P2***		0	8.84	8.16	8.16
	***(bottom)***	***P1***	0	9.92	8.12	7.40	7.10

**Table 3:** Root mean square error (*RMSE*) scores on the fidelity/distortion judgment between laser-induced acoustic responses for different contents in a PP container as a function of the sampling position.

*RMSE*				
	*Sampling Point*	*Contents*		
		*Air-Water*	*Air-Sand*	*Water-Sand*
***(top)***	***P5***	6.99	6.55	5.74
	***P4***	8.38	8.76	5.92
***(center)***	***P3***	7.28	8.75	7.10
	***P2***	8.28	8.30	8.46
***(bottom)***	***P1***	12.66	9.62	8.90
